# Microsatellite and Mitochondrial DNA Study of Native Eastern European Cattle Populations: The Case of the Romanian Grey

**DOI:** 10.1371/journal.pone.0138736

**Published:** 2015-09-23

**Authors:** Daniela Elena Ilie, Ada Cean, Ludovic Toma Cziszter, Dinu Gavojdian, Alexandra Ivan, Szilvia Kusza

**Affiliations:** 1 Department of Research, Research and Development Station for Bovine, Arad, Arad, Romania; 2 Department of Animal Production Engineering, Banat’s University of Agricultural Sciences and Veterinary Medicine 'King Michael I of Romania', Timisoara, Timiș, Romania; 3 Department of Research, Research and Development Station for Sheep and Goats, Caransebes, Caraș-Severin, Romania; 4 Department of Functional Sciences, University of Medicine and Pharmacy "Victor Babes" Timisoara, Timiș, Romania; 5 Animal Genetics Laboratory, Institute of Animal Science, Biotechnology and Nature Conservation, University of Debrecen, Debrecen, Hungary; Wageningen UR Livestock Research, NETHERLANDS

## Abstract

The Eastern European Grey cattle are regarded as the direct descendants of the aurochs (*Bos taurus primigenius)*. Nowadays in Romania, less than 100 Grey animals are being reared and included in the national gene reserve. We examined the genetic diversity among Romanian Grey, Brown, Spotted and Black and White cattle breeds, with a particular focus on Romanian Grey through the use of (i) 11 bovine specific microsatellite markers on 83 animals and (ii) 638 bp length of mitochondrial DNA (mtDNA) D-loop region sequence data from a total of 81 animals. Both microsatellite and mtDNA analysis revealed a high level of genetic variation in the studied breeds. In Romanian Grey a total of 100 alleles were found, the mean number of observed alleles per locus was 9.091; the average observed heterozygosity was 0.940; the Wright’s fixation index (F_IS_) was negative (-0.189) and indicates that there is no inbreeding and no selection pressure. MtDNA analysis revealed 52 haplotypes with 67 variable sites among the Romanian cattle breeds without any insertion or deletion. Haplotype diversity was 0.980 ± 0.007 and ranged from 0.883 ± 0.056 (Brown) to 0.990 ± 0.028 (Spotted and Black and White). The highest genetic variability of the mtDNA was recorded in the Grey breed, where 18 haplotypes were identified. The most frequent mtDNA D-loop region belonged to T3 haplogroup (80.247%), which was found across all studied breeds, while T2 haplotypes (16.049%) was only found in Grey, Spotted and Black and White genotypes. The T1 haplotypes (3.704%) were found in the Grey and Spotted. The current results contribute to the general knowledge on genetic diversity found in Eastern European cattle breeds and could prove a valuable tool for the conservation efforts of animal genetic resources (FAnGR).

## Introduction

Cattle are an important livestock species that have played a special role in the human history and culture, and had a considerable impact on human society. The worldwide population of cattle is estimated to 1.4 billion animals, of which 159 million (11%) are found in Europe and Central Asia [1,2]. Romania is among the top ten countries in the EU regarding the cattle population with 2,022,400 heads and a breed structure of 30.96% Romanian Spotted, 20.28% Romanian Black and White and 13.58% Romanian Brown, according to National Agency for Animal Improving and Reproduction from Romanian [3].

Currently, in the European countries a total of 464 cattle breeds are classified as either local or regional according to the list of Domestic Animal Diversity [4], however, their numbers should be higher since around 130 cattle breeds have disappeared or are currently under threat of extinction. This decrease in the number of cattle breeds has several reasons, such as modernization and reorientation of the agricultural production, socio-economic changes and cultural developments. Between the 1950’s and 1980’s, the willingness to increase productivity, intensification and specialization of animal production has dramatically affected the local cattle breeds and resulted in loss of sequence variation in DNA and breeds diversity [5,6]. However, in the last two decades the interest for preserving the locally adapted breeds has considerably increased and numerous conservation strategies were implemented in Europe and worldwide. Therefore, several studies on *Bos taurus* mtDNA were carried out and revealed three predominant and geographically structured taurine haplogroups: T1 found in Africa; T2 originates in the Near East and Western Asia; and T3 found in Europe [7,8] and originates from the expansion of a small cattle population domesticated in the Middle East [9]. Strong geographic differentiation of cattle mtDNA suggests strong founder effects during the earliest migrations of cattle, which was present only in a few individuals [10].

Autochthonous cattle breeds, important in agriculture, are considered historical heritage and valuable genetic resources due to their adaptation to local conditions and the unique genetic material, aspects that are important for breeding and conservation programmes. The Romanian Grey is a valuable reservoir of genes with high importance for the Romanian agriculture. Studies conducted on the current population of Romanian Grey from Moldavia (Dancu—Iasi) outlined the low milk production potential of the breed, which ranged between 1589 kg in the first lactation and 2535 kg in the 5^th^ lactation, with a content of 4.71% fat and 3.71% protein [11]. Despite these low productions the most important characteristics of the breed are: high longevity, adaptability, hardiness, resistance to diseases, high resistance to extreme temperatures, high fat and protein content of milk [12].

The Romanian representatives for Food and Agriculture Organization (FAO) classified the Romanian Grey as endangered and in urgent need for *in situ* conservation [13]. The Romanian Grey is regarded as a direct descendant of the *Bos taurus primigenius* and is included in the South-Estern European Podolian breed group (found in Bulgaria, Croatia, Greece, Hungary, Italy, Romania, Turkey, Ukraine and Serbia). There is a small population of local Grey cattle in Estonia as well, which appear, however, to be a mix of old native cattle stock and modern breeds [14]. The origin of Grey cattle is not well defined and there are different hypothesis. It is supposed that ancestral animal may have come from Mediterranean areas or from Asia. One of the most accepted hypothesis is that Europe was a domestication center for modern cattle from wild aurochs [15], while the second one presents the South-Eastern basin as an entry point for cattle population from Asia [16]. Genetic pattern of zebu was detected by microsatellite markers in the Podolian cattle, and appeared to originate from ancient Steppe cattle which expanded to Central-Europe from Russian southern steppe more than 1000 years ago [17–19]. Until 1850 the Romanian territory was populated only by autochthonous breeds of *Bos taurus primigenius* descent [11], namely Romanian Grey and Mocanita. Their number was about 1,860,726–2,607,594 at that time and most of them belonged to Romanian Grey breed [16]. The breed was divided in five ecotypes named after the geographical area of formation: *Transylvanian*, *Moldavian*, *Bucsan*, *Ialomițean* and *Dobrogean* [11,20,21]. The number of Romanian Grey cattle registered a dramatic decline after the World War I recording a number of 200 heads in 1996 [21]. Currently, the breed is listed as endangered, being raised in a very low number of animals (< 100) in Iași, Danube Delta and Piatra Neamț [11,20,22], and was therefore included into a national genetic conservation programme.

The aim of the current study was to evaluate the genetic diversity among Romanian Grey, Brown, Spotted and Black and White cattle breeds with a particular focus on the endangered Romanian Grey breed, in order to provide information for future breeding programmes and conservation management strategy of the breed. We investigated the above mentioned cattle breeds using the analysis of 11 microsatellite loci and the D-loop region of mtDNA. In addition, a comparative analysis between the current investigated cattle breeds and that of 525 mtDNA from GenBank was performed in order to reveal genetic relationships of the studied breeds with other breeds.

## Material and Methods

### Animals and extraction of DNA

**Fig 1:**
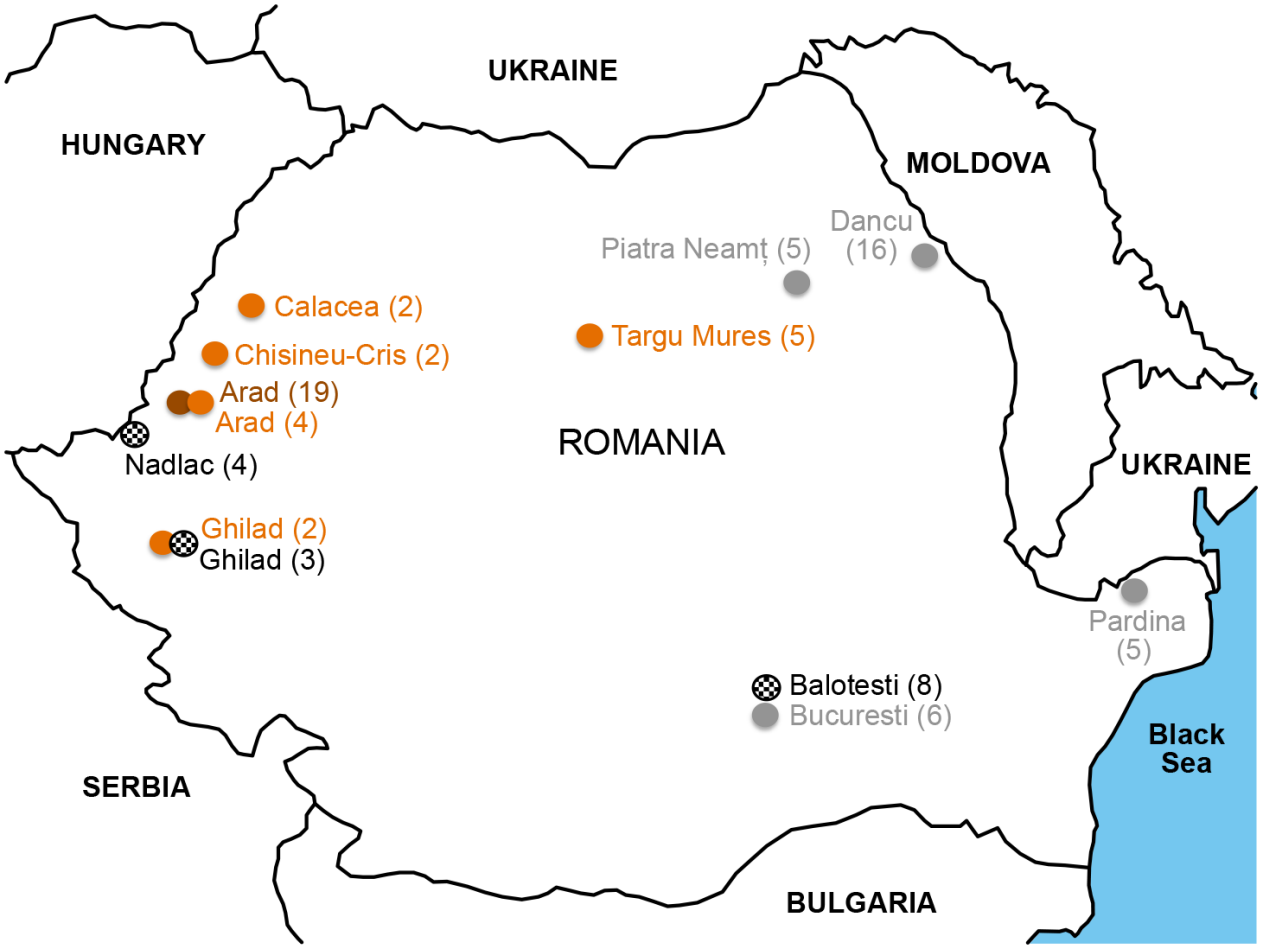
Sampling site and numbers (in parentheses) of the samples (colours: grey—Romanian Grey; brown—Romanian Brown; orange—Romanian Spotted; black and white- Romanian Black and White).

Altogether 93 individuals belonging to different breeds were included in the study: Romanian Grey (N = 32; 6 males and 26 females), Romanian Brown (N = 19 females), Romanian Spotted (N = 15; 5 males and 10 females), Romanian Black and White (N = 15; 1 male and 14 females) and German Spotted (N = 12 males). The animals were unrelated and were randomly selected from different herds and geographical regions in order to avoid genetic similarities (Fig 1). The collection sites and geographic coordinates of the Romanian breeds used in the study were given in S1 Table. Romanian Grey females samples were collected from three sites (Fig 1): Dancu—Iași (N = 16 females), Piatra Neamț (N = 5 females) and Pardina—Tulcea (N = 5 females), while the male samples (N = 6) were provided by the National Agency for Animal Improving and Reproduction from Romania.

Total DNAs were extracted from blood and semen. Fresh blood (2 ml) was collected from the tail vein (*vena cava*) of the animals in vacutainers containing K3EDTA as anticoagulant (Vacutest Kima, Italy). Authorized veterinarians performed collection of blood samples. During the blood collection the animals were kept confined as to prevent injuries. Genomic DNA was isolated from 300 μl blood samples using a commercial DNA extraction kit (Wizard Genomic DNA Purification Kit, Promega, Madison, USA), according to manufacturers’ instructions.

For isolation and purification of DNA from semen a classical method of extraction was used. The principle of the method consisted of the seminal cell lysis for at least 6 hours in lysis buffer (1M Tris pH 8.0; 5M NaCl; 0.5 M EDTA pH 8.0; 10%SDS), 50 μl DTT 500 mM and 10 μl protease K (20 mg/ml), protein precipitation with a 6M NaCl solution and extraction of the total genomic DNA by precipitation with 70% ethanol and isopropanol solution, followed by rehydration of the DNA in TE (TRIS 10 mM pH 7.4 and EDTA 1 mM). DNA concentration was evaluated spectrophotometrically, with NanoDrop-2000 (Thermo Fisher Scientific Inc., USA), and visually by standard agarose gel electrophoresis (1% agarose (w/v) in TBE).

### Ethics statement

The research activities were performed in accordance with European Union’s Directive for animal experimentation (Directive 2010/63/UE). The experimental design, sampling collection protocols and procedures were approved by the Ethics Committee of the Research and Development Station for Bovine Arad, throughout the Decision no.14/06.12.2012.

### Microsatellite analysis

All animals were genotyped with a panel of eleven microsatellite loci: TGLA227, BM2113, TGLA53, ETH10, SPS115, TGLA126, TGLA122, INRA23, ETH3, ETH225 and BM1824, respectively. These loci are recommended by the International Society for Animal Genetics (ISAG) / Food and Agriculture Organization of the United Nations (FAO) Advisory Committee for genetic diversity studies [23] for cattle genetic diversity analysis. All of the microsatellite markers were amplified simultaneously by multiplex PCR in a single reaction. Amplification was realized using PCR primers labeled with different fluorescent dyes (JOE, NED, FAM) using StockMarks for Cattle Bovine Genotyping Kit (Applied Biosystems, CA, USA) according to reaction protocols of ABI that were followed. As a reference we used the DNA control provided by the manufacturer. The Genescan^®^ Analysis software (Applied Biosystems) was used to determine the PCR fragments sizes by comparison with internal size standards *Rox*.

#### Data analysis

ARLEQUIN v.3.1. [24], GENEPOP v.4.2. [25,26] and FSTAT v. 2.9.3.2 [27] was used to estimate basic population genetic descriptive statistics for each microsatellite marker: number of alleles per locus, the observed heterozygosity (H_O_) and expected heterozygosity (H_E_) according to Levene [28]. Breed genetic structure was analyzed using Wright’s fixation index [29]. Genetic diversity was assessed by effective population size (Ne) and it was calculated by linkage disequilibrium method, as implemented in the software package NeESTIMATOR v. 2.01 [30]. Deviations from Hardy—Weinberg equilibrium (HWE) were tested for each breed and each locus using the Markov chain method implemented by Guo & Thompson (1992) [31], using the software Genepop [25]. Parameters of the Markov chain expressed as dememorizations/batches/iterations were 10 000/100/5000. Assignment test [32] was used to assign the individuals to the population from which they were actually sampled by simulating them 10000 times per samples. MICRO-CHECKER v. 2.2.3 [33] was used to estimate null allele frequency for each locus using methods by Chakraborty et al. [34] and Brookfield et al. [35]. Polymorphic information content (PIC) values were estimated using CERVUS v. 3.0 [36]. Sign, standardized differences and Wilcoxon sign rank tests under three models (IAM—infinite allele model, SMM—stepwise-mutation model, TPM—two-phased model) and mode shift test were performed with BOTTLENECK v.1.2.02 [37] in order to detect genetic bottleneck signatures. MEGA v 6.05 [38] was used to construct neighbour-joining (NJ) tree based on pairwise F_ST_ values to estimate phylogenetic relationships among breeds. In addition, the Factorial Correspondence Analysis (FCA) was performed to visualize the relationships, possible admixtures among the individuals from different breeds using GENETIX v. 4.05.2 [39].

### Mitochondrial DNA analysis, mtDNA amplification and sequencing

Amplification was carried out by using primers designed to amplify an 825 bp fragment length from the D-loop of mitochondrial DNA (mtDNA) between positions 103 and 927 (accession number U92243), based on the published *Bos taurus* mitochondrial D-loop DNA reference sequence [40]. The two flanking primers used were as follows: forward: 5’-CAGAATTTGCACCCTAACCAA-3’ and reverse: 5’-GGGGCCTGCGTTTATATATTG-3’. Polymerase chain reaction (PCR) was performed in a 10 μl reaction mixture containing 60–80 ng of genomic DNA, 0.1 mM dNTP, 2 mM MgCl_2_, 0.7 U of GoTaq DNA Polymerase, 5X Colorless GoTaq Reaction Buffer (Promega, USA) and 10 pmol of each primer. PCR conditions were as follows: 10 min at 95°C, 35 cycles of 30s at 95°C, 45 s at 60°C, 30s at 72°C and a final step at 72°C for 10 min. Sequencing reactions were performed by Macrogen Inc. (The Netherlands)

#### Data analysis

Sequences were aligned using CLUSTALW [41], and manually checked in the BIOEDIT program [42]. Haplotype and nucleotide diversity, number of polymorphic sites, average number of nucleotide differences were calculated with DnaSP version 5.10 [43]. ARLEQUIN v. 3.1. [24] was used for calculation of past population expansion (both Tajima’s D test, Fu’Fs test) [44,45] and demographic expansion (Harpending raggedness index (r) and SSD and reflect the shape of mismatch distribution). In case of neutrality tests (Tajima's D and Fu's Fs) the significant and negative values, while nonsignificant SSD and r values are support of population expansion. Smooth, unimodal distributions can be regarded as being representative of a population expansion, while the ragged and multimodal mismatch distributions indicate a stationary population [46].

In addition, networks were also calculated using a median-joining approach with default settings using NETWORK v. 4.111 [47]. Networks suit more to depict intraspecific phylogenies than tree algorithms because they allow the co-existence of ancestral and descendant alleles in a sample [48].

## Results

### Genetic variability at 11 microsatellite loci

Eleven bovine microsatellite markers were successfully amplified in 83 animals from the five breeds: Romanian Grey (N = 29), Romanian Brown (N = 16), Romanian Spotted (N = 13), Romanian Black and White (N = 13) and German Spotted (N = 12). A total of 124 alleles were found at the eleven loci in the 83 animals belonging to five breeds. In this study we focused on Romanian Grey, therefore diversity indexes for the Grey breed are presented in Table 1. The same indexes for the other breeds are available from corresponding author by request. PIC values indicate the information of the microsatellite loci studied. In the present study the PIC values for all the 11 microsatellite loci ranged from 0.523 (SPS115) to 0.863 (TGLA122). The total number of alleles was 100, with an average of 9.091 alleles per locus, ranging from 5 (ETH225) to 15 alleles (TGLA122). Significant deviations from the HWE (P<0.001; P<0.05) were observed at ETH225, BM2113, TGLA122, TGLA53 and SPS115 loci. The mean H_E_ value was 0.794, with a minimum value of 0.574 (SPS115) and a maximum value of 0.890 (TGLA122). The observed heteorozygosity (H_O_) values were ranging from 0.552 (SPPS11) to 1.000 (ETH3, BM2113, BM1824, TGLA227, TGLA126, TGLA53).

**Table 1:** Allele size range, polymorphic information content (PIC), observed number of alleles (n_O_), expected (H_E_) and observed (H_O_) heterozygosity at 11 loci in Romanian Grey. The highest and lowest values are in bold.

Marker name	Allele size range	n_O_	H_O_	H_E_	PIC
ETH225**	136–165	**5**	0.966	0.712	0.645
INRA23	193–235	9	0.966	0.845	0.810
ETH10	198–234	8	0.897	0.799	0.753
ETH3	90–135	9	**1.000**	0.831	0.792
BM2113*	116–146	8	**1.000**	0.823	0.784
BM1824	170–218	7	**1.000**	0.788	0.737
TGLA227	64–115	13	**1.000**	0.811	0.775
TGLA126	104–131	8	**1.000**	0.786	0.744
TGLA122*	134–193	**15**	0.966	**0.890**	**0.863**
TGLA53*	147–197	11	**1.000**	0.872	0.842
SPS115*	235–265	7	**0.552**	**0.574**	**0.523**
Mean		9.091 ± 2.745	0.940 ± 0.127	0.794 ± 0.083	0.752

*P ≤ 0.05,

**P ≤ 0.001

The mean expected heterozygosity ranged from 0.755 (Romanian Spotted) to 0.794 (Romanian Grey), while the observed heterozygosity ranged from 0.895 (Romanian Spotted) to 0.966 (Romanian Brown), (Table 2). A heterozygote deficiency was not observed for any of the studied loci. In fact, the null allele test revealed that no loci show evidence for a null allele. The heterozygote excess can be pointed out with the values of F_IS_. The global F_IS_ was estimated to -0.197 and was not significantly different from zero (P < 0.001).

**Table 2:** Number of animals, observed number of allele (n_O_), observed (H_O_) and expected (H_E_) heterozygosity and Wright’s fixation index (F_IS_) in all investigated Romanian cattle breeds. The highest and lowest values are in bold.

Population	n	Observed number of allele (no) (s.d.)	Ho	He (s.d.)	F_IS_
**Romanian Grey**	29	**9.091** ± 2.745	0.940 ± 0.127	**0.794** ± 0.083	-0.189
**Romanian Brown**	16	7.182 ± 2.037	**0.966** ± 0.090	**0.788** ± 0.065	-0.235
**Romanian Spotted**	13	**6.909** ± 2.275	**0.895** ± 0.183	0.755 ± 0.125	-0.194
**Romanian Black and White**	13	7.455 ± 2.271	0.930 ± 0.155	0.779 ± 0.102	-0.203

The estimated mean Ne for the Romanian Grey population was 37.8 (95% CIs = 25.0–67.2), 31.7 (95% CIs = 23.7–45.4) and 46.3 (95% CIs = 34.2–68.3) under different excluded allele frequencies: <5%, <2% and <1%, respectively. LDNe estimated finite estimates only in that breed.

The calculated F_ST_ values indicate that around 4.5% of the total genetic variation could be explained by differences between breeds and the remaining 95.6% may correspond to differences between individuals.

**Fig 2:**
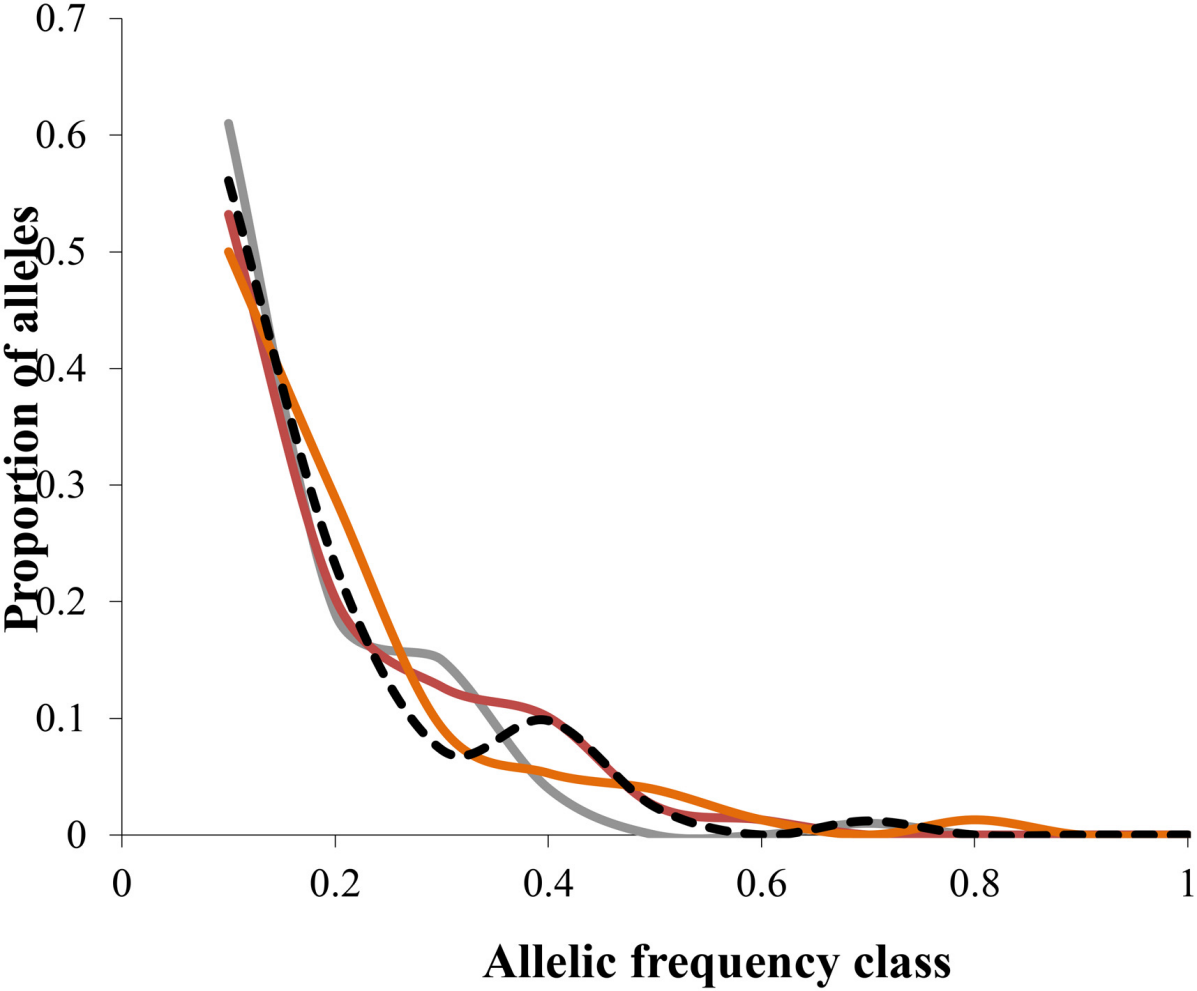
Distribution of a proportion of alleles in different allelic frequency classes. Colours represent breed: grey—Romanian Grey; brown—Romanian Brown; orange- Romanian Spotted; black and white—Romanian Black and White.

Microsatellite data was also used to evaluate potential recent bottleneck events within breeds. The expected number of loci with heterozygosity excess was 6.57, 6.49 and 6.49 for IAM, TPM and SMM, respectively. The null hypothesis was not rejected using the Sign test and not indicated a recent genetic bottleneck event. In case of standardised difference test, the hypothesis of mutation-drift equilibrium was rejected only for SMM (*P* = 0.001) model. The Wilcoxon test, which gives high statistical accuracy and can be used for a low number of polymorphic loci and any number of individuals, also indicated an excess in heterozygosity and bottleneck in one model with probability values of 0.002 (IAM). Mode-shift indicator test as a second test for potential bottleneck was used. The allelic class and proportion of alleles showed a normal „L” shaped distribution for all studied breeds (Fig 2).

Pairwise estimates of F_ST_ were calculated between pairs of Romanian breeds and German Spotted (Table 3). This breed was included in the study as outgroup because it is relatively close to the Romanian breeds excepting the Romanian Grey. All pairwise F_ST_ values were significantly different from zero (P<0.05). The highest genetic differentiation was found between Romanian Brown and the Romanian Spotted, with pairwise F_ST_: 0.381 and Romanian Brown and Romanian Black and White (0.257). Romanian Grey had the least genetic differentiation from the Romanian Spotted (0.031).

**Table 3:** Population pair-wise differentiation according to F_ST._

Population	Romanian Grey	Romanian Brown	Romanian Spotted	Romanian Black and White	German Spotted
**Romanian Grey**					
**Romanian Brown**	0.040				
**Romanian Spotted**	0.031	0.381			
**Romanian Black and White**	0.041	0.257	0.024		
**German Spotted**	0.048	0.070	0.011	0.050	

Based on the result of an assignment test, 30.77% (Romanian Spotted) to 81.25% (Romanian Brown) of individuals were correctly assigned to their breeds (Table 4).

**Table 4:** Assignment test.

Population	n	N	%
**Romanian Grey**	29	7	75.86
**Romanian Brown**	16	3	81.25
**Romanian Spotted**	13	9	30.77
**Romanian Black and White**	13	6	53.85
**German Spotted**	12	4	66.67

n = number of tested animals

N = number of animals incorrectly assigned

% = percentage of animals correctly assigned into their group

**Fig 3:**
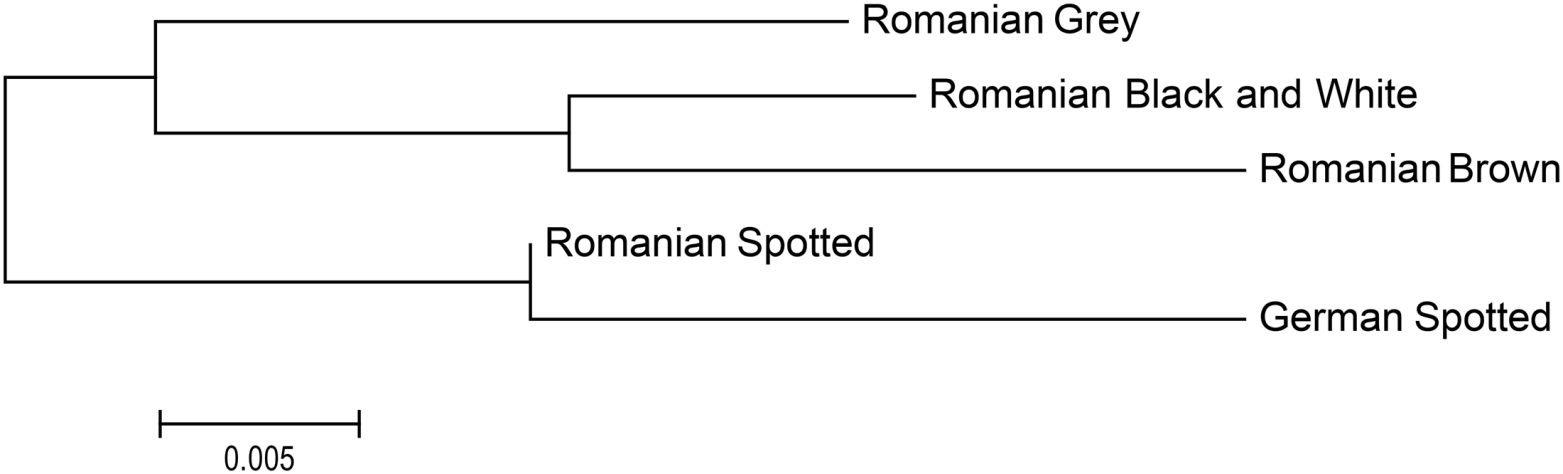
Neighbour-joining phylogenetic tree showing genetic relationships among the five cattle breeds.

A phylogenetic tree was constructed using the neighbour—joining (NJ) method based on F_ST_ genetic distance values (Fig 3) and revealed that the clustering of the examined breeds was consistent with the current knowledge of their history and breed formation (Fig 3).

**Fig 4:**
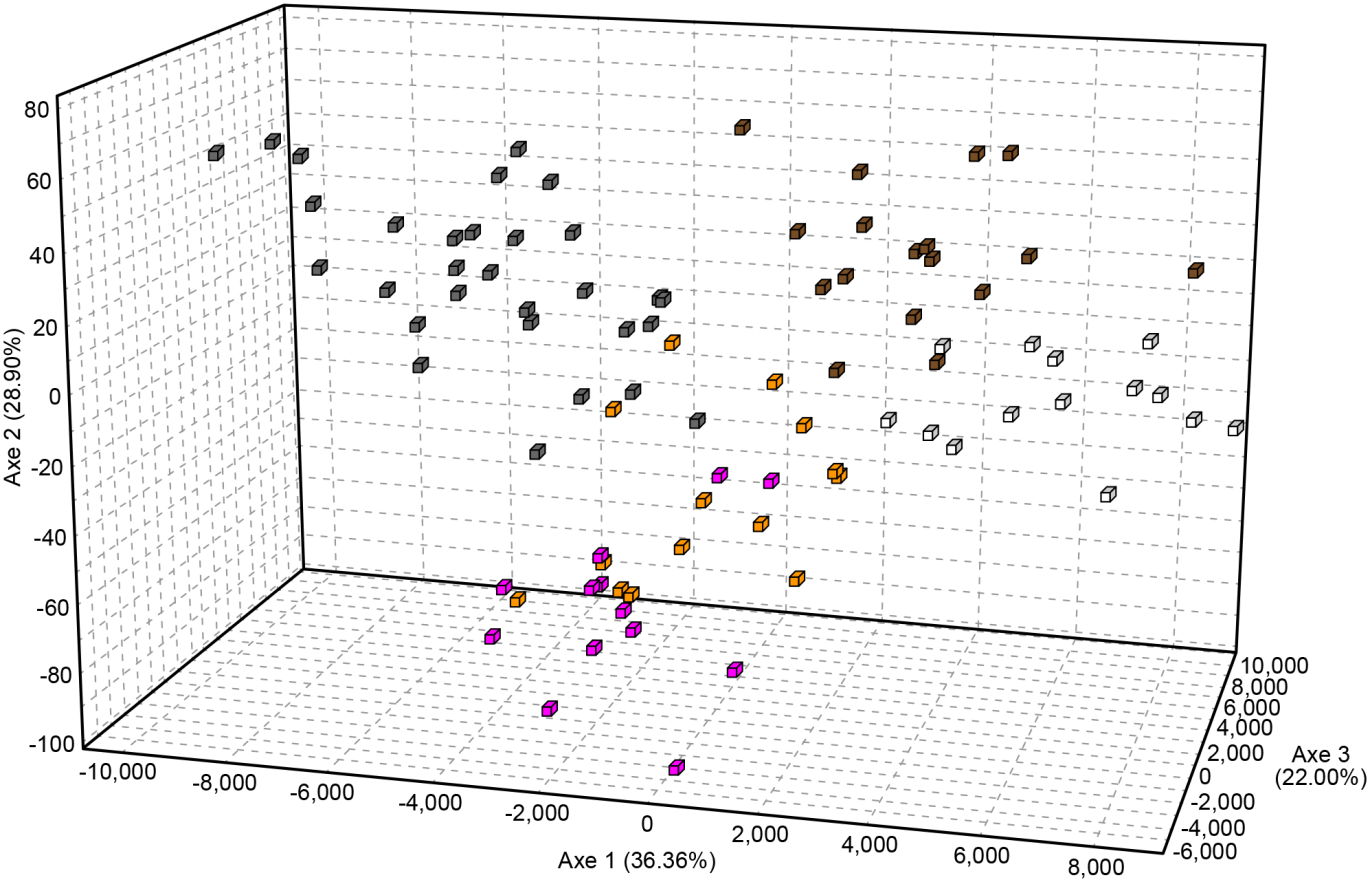
Three-dimensional factorial correspondence analyses (3D FCA) of five studied cattle breeds based on 11 microsatellite loci. The axes 1–3 explain the percentage of the variance among the breeds. Colours indicate the following breeds: grey- Romanian Grey; pink- German Spotted; orange—Romanian Spotted; brown- Romanian Brown; white—Romanian Black and White.

Similar clustering results were obtained for phylogenetic relationship of the individuals from the studied breeds by factorial correspondence analysis (Fig 4). The first three components accounted for 87.3% of the variance of the data. Approximately, 33, 22 and 29% of the total variance was explained among the three components. The three dimensional FCA shown that Romanian Grey is separated from the other studied breeds, while Romanian Black and White admixtured with Romanian Brown and individuals from Romanian Spotted were mixed with German Spotted, as it was expected.

### Genetic variability at D-loop region of mitochondrial DNA

After aligning and trimming the sequences of 32 Romanian Grey, 19 Romanian Brown and 15–15 Romanian Spotted and Romanian Black and White with the fragment size of 638 bp were further analysed. The haplotypes, haplogroups and variable nucleotide positions observed in Romanian cattle breeds are presented in relation to the reference sequence V00654 [49] from GenBank (S2 Table).

Among the 81 samples of the Romanian breeds, 52 haplotypes (GenBank accession numbers: KR608070-KR608121) were detected by 67 polymorphic sites (S2 Table). Eighteen haplotypes were detected in Romanian Grey, 10 in Romanian Brown and 14–14 in Romanian Spotted and Black and White breeds, respectively (Table 5). High number (37) of singleton haplotype was detected in all studied breeds. Only 3 haplotypes were shared among the breeds. Haplotype 6 contained 4 animals, 1 from each breed, haplotype 10 merged one Romanian Spotted and one Romanian Black and White, while the haplotype 51 had one animal from Romanian Grey and one animal from Romanian Brown breeds.

**Table 5:** Basic parameters of genetic (mtDNA) variability and values of neutrality tests (Fs and D) of Romanian cattle breeds.

	No. of individuals	No. of haplotypes	No. of polymorhic sites (S)	Haplotype diversity h±s.d.	Nucleotide diversity π±s.d.	Fu’s *Fs*	Tajima's D
**Romanian Grey**	32	18	27	0.927 (0.033)	0.008 (0.001)	-6.139*	-0.901
**Romanian Brown**	19	10	20	0.883 (0.056)	0.007 (0.001)	-1.462	-0.859
**Romanian Spotted**	15	14	22	0.990 (0.028)	0.006 (0.001)	-10.469**	-1.675*
**Romanian Black and White**	14	14	24	0.990 (0.028)	0.007 (0.001)	-10.033**	-1.752*
**Total**	81	52	67	0.980 (0.007)	0.008 (0.000)	-25.575**	-2.114*

*P ≤ 0.05,

**P ≤ 0.001

As expected for the European cattle breeds, the T3 haplogroup had the highest frequency (80.247%). A total of 65 animals were assigned to the T3, thirteen to the T2 (16.049%) and three to T1 (3.704%) (S2 Table). The T3 haplotypes were found across all studied breeds, whereas T2 haplotypes (N = 13) were only found in the Grey (10/13), Spotted (2/13) and Black and White (1/13) breeds. The T1 haplotypes (N = 3) were found in the Grey (2/3) and Spotted (1/3) breeds.

Haplotype diversity was 0.980 ± 0.007, ranged from 0.883 ± 0.056 (Romanian Brown) to 0.990 ± 0.028 (Romanian Spotted and Romanian Black and White). Nucleotide diversity values were also high in all studied Romanian breeds.

**Fig 5:**
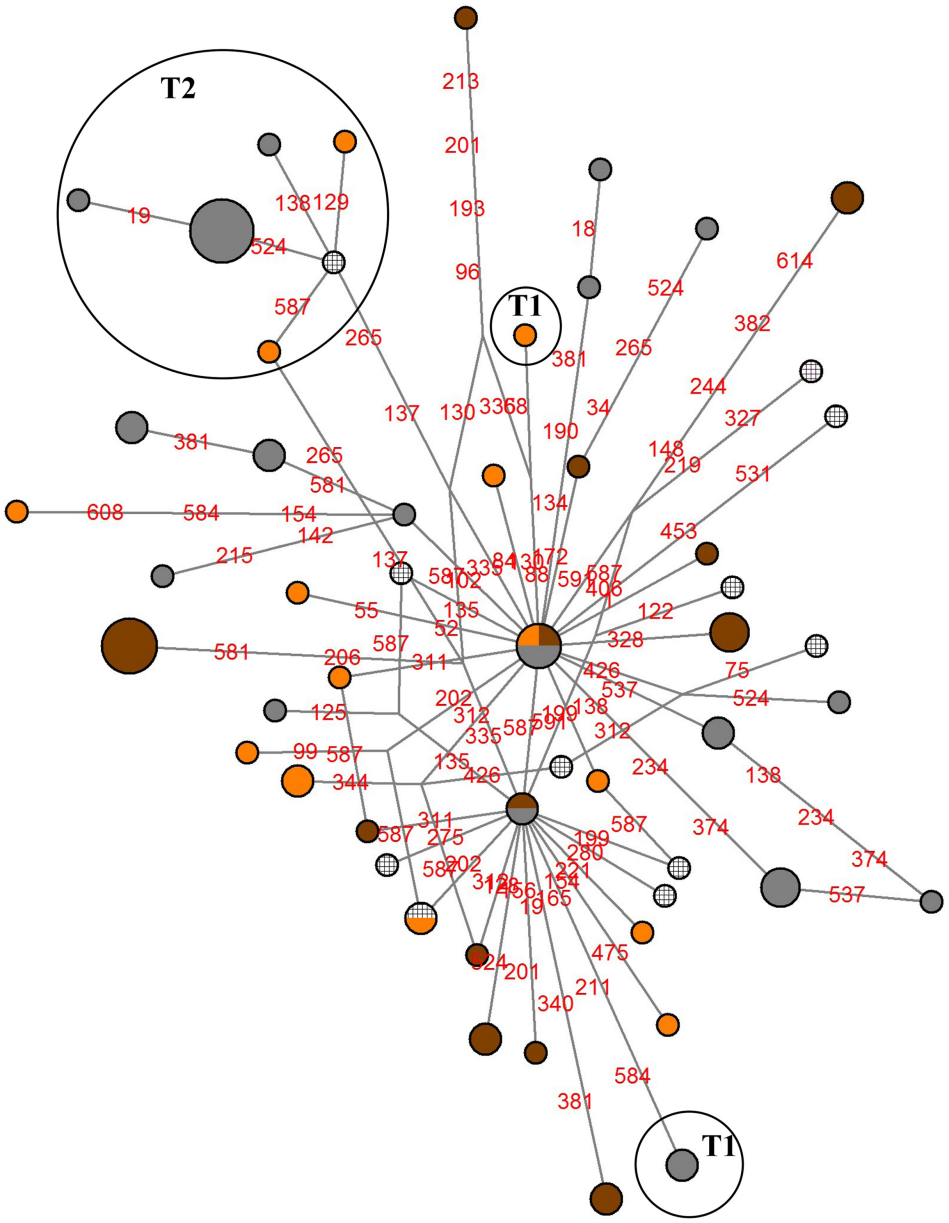
Median-joining network of 81 Romanian animals based on the mtDNA sequences. The size of the circle is proportional to the number of animals represented. Colours represent breed: grey—Romanian Grey; brown—Romanian Brown; orange- Romanian Spotted; black cross—Romanian Black and White. Numbers on the lines indicates the number of mutations.

Median-joining network of 52 haplotypes was constructed (Fig 5) and showed the high number of singleton haplotypes. There are only three shared haplotypes among breeds.

**Fig 6:**
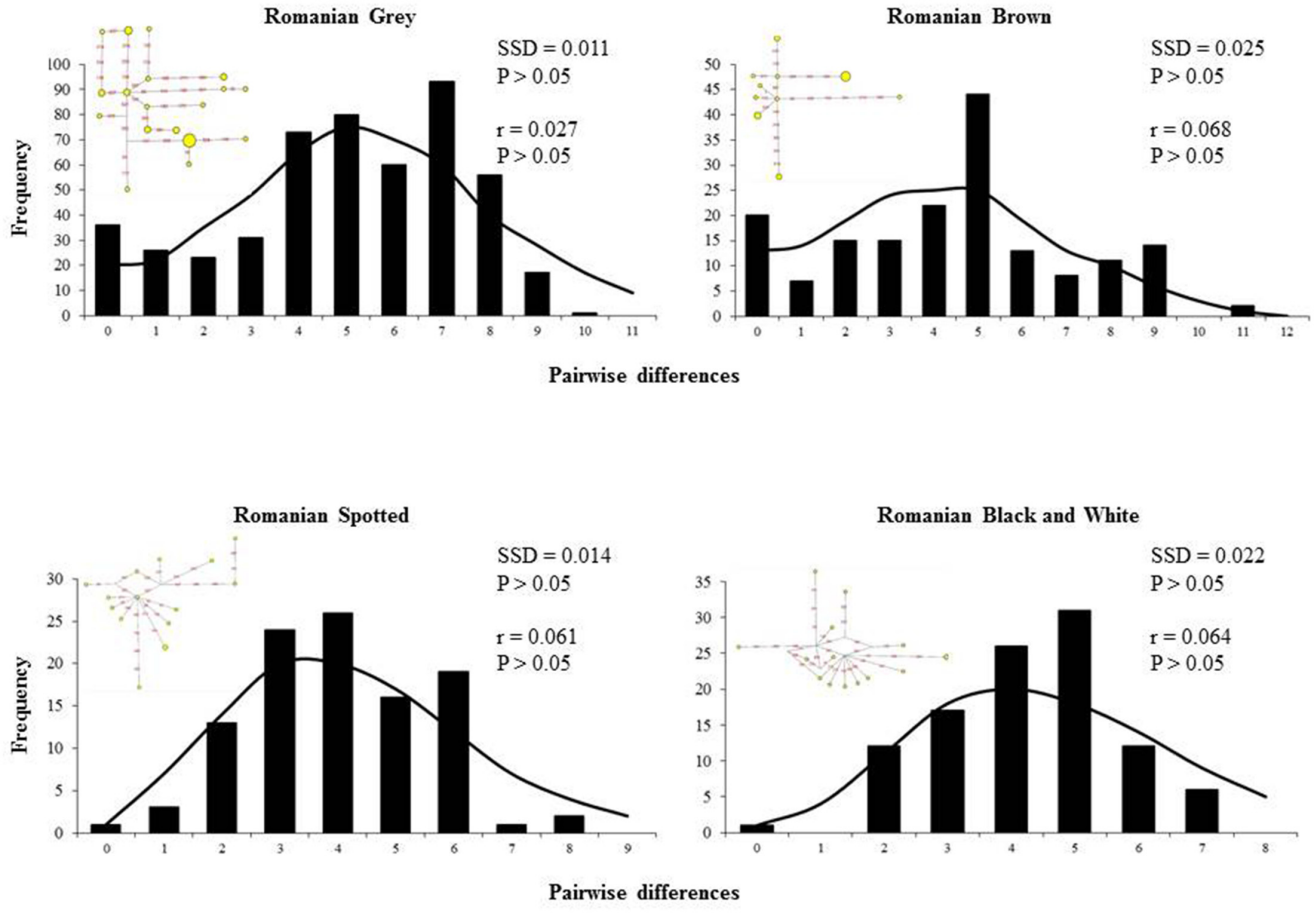
Mismatch distributions based on pairwise site differences between sequences. The expected curve (solid line) was obtained from simulated values computed from the data under the model of demographic expansion.

The mismatch distribution in Romanian Black and White breed was only smooth and unimodal (Fig 6). Expansion hypothesis was further supported by negative and significant Fu’s and Tajima’s D neutrality tests (FS = 10.033, P ≤ 0.001; D = -1.752, P ≤ 0.05) (Table 5). In the case of the other breeds the shape of the distribution was ragged and multimodal. Fu’s *Fs* test, which is based on the distribution of haplotypes, was negative and significantly different from zero for all breeds, except Romanian Brown. Nonsignificant Harpending's raggedness index and sum of square deviations (SSD) indicates a good fit and support the expansion. SSD values were not significant and Harpending’s raggedness indexes were also positive and non-significant, indicating a good support of past expansion (Fig 6). The median-joining networks among haplotypes did not revealed a star-like distribution trend with one central positioned haplotype in any breed.

Our sequences were aligned with 525 mtDNA cattle sequences with fully overlapping standard 379 bp in length obtained from the GenBank database (S3 Table). The Romanian breeds were not clearly separated from other breeds in all cases and gave unexpected results. Geographical or breed distribution was not found among the breeds. In Romanian Grey 12 haplotypes were detected that share the same haplotype with Hungarian Grey in two cases, while in another case share haplotypes with Podolian cattle (Hungarian Grey, Bulgarian Grey, Ukrainian Grey, Istrian and Slavonian Syrmian), Brahman breed (*Bos indicus*) and Portuguese and Spanish breeds (Maronesa, Ramo Grande and Palmera).

## Discussion

### Risk of genetic diversity loss in Romania

Current results show that there is a large genetic diversity among and within the Romanian local cattle breeds. However, some breeds are at risk because of the human activities such as: pollution, agriculture intensification, overexploitation of natural resources etc. Romania has a unique geographical localization since it is part of the Carpathian basin and Balkanic region, and has been affected by the different neighbouring biogeographical regions, while maintains a significant genetic reservoir for several domestic species: Mangalica and Bazna pigs, Carpatina and Banat’s White goats, Transylvanian naked-necked chicken, Hucul horse, Turcana and Racka Zackel sheep, the diverse Tsigai sheep group, Transylvanian and Palas Merino, Romanian buffalo etc. and wild animal species: brown bear, wolf, European hare, wild boar, etc. [21,50–57].

Genetic diversity of cattle that is being lost or at risk is of great value to the agricultural, economic, social and cultural sectors. From that point of view every country must take action to conserve the local cattle breeds which are better adapted to their environment and are important for their unique genetic resource and to prepare conservation and breeding strategies for all endangered breeds and populations. However, these actions are impossible without the knowledge of the genetic diversity of the existing breeds and populations. There is a few number of genetic studies about Podolian cattle [6,19,58–62], however, to the best of our knowledge, only Georgescu et al. [50] and Xuan et al. [51] performed analysis on the Romanian Grey and only on individuals from one sampling site. As a result, the current study gives the first information about the Romanian Grey from such a large sample size (comparing to the existing breed size) and from all three geographical regions where the breed exists.

### Genetic variability of Romanian cattle breeds based on 11 microsatellite loci

For the investigated breeds a number of 124 alleles were detected at the 11 studied loci. The highest number of alleles for all investigated breeds was founded at TGLA122 locus (20). At this locus an emphasized genetic difference among the studied breeds was found. Thereby, 13 alleles were detected in Italian Podolian cattle, 26 in Turkish cattle, 11 in Slavonian Syrmian Podolian cattle and 23 in Croatian Podolian cattle [61–64]. For the loci ETH10, BM1824 and SPS115, Keros et al. [61] found a higher numbers of alleles (10, 11 and 12, respectively) in the Slavonian Syrmian Podolian cattle, whereas only 8 alleles were observed at the same loci in our study.

The average number of alleles per locus in Romanian Grey cattle was of 9.091, higher than reported in other Podolic cattle, such as in Italian Podolica (8.5), Bulgarian Grey (7.6) [65,62] and lower than in Istrian cattle (12.55) [64]. A study carried out by Georgescu et al. [50] on Romanian Grey cattle (N = 30) from a restricted geographic area, where the same 11 loci were analyzed, showed lower mean number of alleles per locus (6.36) than in the current study. This discrepancy can be explained by the fact that the investigations of the present study were conducted on existing Romanian Grey cattle from all the three geographic areas: Iași, Danube Delta and Piatra Neamț. The lowest number of alleles was found in ETH225 locus (5), value that is in concordance with results obtains by Georgescu et al. [50]. The small number of alleles at this locus in Romanian Grey might be the result of a low number of investigated animals. However, by analyzing the results of the present study and comparing them with the results obtained on Romanian Grey breed by Georgescu et al. [50], high number of alleles for the rest of the loci (INRA23, ETH10, ETH3, BM2113, BM1824, TGLA227, TGLA126, TGLA122, TGLA53 and SPS15) were observed (9, 8, 9, 8, 7, 13, 8, 15, 11 and 7; 8, 6, 7, 8, 5, 5, 7, 5, 8 and 6, respectively).

The Romanian Grey breed showed a high level of mean expected (0.794 ± 0.083) and observed (0.940 ± 0.127) heterozygosity values. At the TGLA122 locus the observed heterozygosity was, 0.740 in Italian Podolian cattle, 0.570 in Slavonian Syrmian Podolian cattle and 0.644 in Austrian and Hungarian cattle breeds [60,61,66]. The observed heterozygosity was also higher (0.940) than those reported for Italian Podolian breed (0.73), Slavonian Syrmian Podolian cattle (0.70), Bulgarian Grey (0.78) and Hungarian Grey (0.658) [60,61,65,66]. In a previous study on Romanian Grey breed conducted on the same 11 loci, the values of observed (0.687) and expected (0.763) heterozygosity were also lower [50].

Another parameter analyzed in Romanian Grey breed was the polymorphism information content (PIC). None of the loci showed PIC values lower than 0.5. PIC of all 11 loci in Romanian Grey breed was quite high with the average of 0.752. Analyzing the individual loci, the highest value for this parameter was observed among TGLA122 (0.863), TGLA53 (0.842) and INRA23 (0.810) loci. In a previous study on Simmental breed conducted on the same 11 loci, the PIC value ranged from 0.58 (ETH10) to 0.88 (TGLA53) with the average of 0.75 [67].

Overall Ho values were higher than He for all investigated breeds. The mean expected (0.788; 0.755; 0.779) and observed (0.966; 0.895; 0.930) heterozygosity for Romanian Brown, Romanian Spotted and Romanian Black and White breeds was found to be higher than those reported for Brown Swiss (0.66; 0.67), Holstein Friesian (0.64; 0.62), Simmental (0.59; 0.69), and Piemontese (0.72; 0.71) [60]. Contrary to a previous study on Romanian cattle breeds, higher genetic variability (H_E_) was also observed in present study [50]. The inbreeding coefficient (F_IS_) for each breed was negative. The negative values of F_IS_, ranging from -0.235 (Romanian Brown) to -0.189 (Romanian Grey) represents an excess of heterozygotes and indicates that there is no inbreeding in all investigated Romanian breeds. The studied breeds point out a high level of genetic variation visible from a high number of alleles per locus. The most genetically variable breed was Romanian Grey.

Bottleneck analysis revealed that the breeds have not undergone any recent bottlenecks, neither any recent reduction in the effective population size, they are in mutation drift equilibrium.

The assignment tests (Table 4), neighbour-joining phylogenetic tree (Fig 3) and Factorial Correspondence Analysis (Fig 4) also showed a genetic similarity between individuals of studied breeds and low level of breed admixture. Romanian Grey breed separated from the other breeds in agreement with the expected results. The other two clusters are as well in agreement with the expected relationship between the breeds. Romanian Spotted and German Spotted have common origins in Simmental cattle group. Furthermore, Romanian Spotted breed has been formed as the result of long-time, non-systematical crossings between the Romanian Grey cows with Simmental-type bulls, imported from different countries (Austria, Germany, Switzerland, Czech Republic and Slovakia). Romanian Brown had the closest relationship to Black and White and it can be explained by the fact that the Romanian Black and White breed was formed as the result of absorption crossing of local breeds such as Brown, Romanian Spotted and especially Dobrogea Red (now extinct) with the Friesian-type bulls of different origins. Previous phylogenetic studies on Romanian cattle breeds have revealed similar level of clustering [50].

### Mitochondrial DNA variability in Romanian cattle breeds

Our study included mtDNA data of Romanian cattle breeds from geographical regions not characterised previously, the Romanian Grey breed considered to be endangered. In spite of few remaining Romanian Grey animals, all three haplogroups T3, T2 and T1 were identified. Analysis of variability of the mtDNA D-loop sequences revealed a high number of haplotypes (52) and polymorphic sites (67) among the 81 animals without any insertion or deletion. In case of Romanian Grey cattle, among 32 animals, 18 haplotypes were identified, indicating a high genetic variability (0.927) of the maternal component of the breed. The detected number of haplotypes in our study was larger than detected in other Podolian-like breeds. In Istrian cattle 50 animals were analysed with 13 haplotypes, in 47 Slavonian Syrmian Podolian cattle 5 haplotypes [58], and in 39 Bulgarian Grey cattle 7 haplotypes were found [68]. Significant lower levels of genetic diversity were observed in Serbian Podolian (0.709; n = 11) and Ukrainian Grey (0.000; n = 8) [19], which could be explained with lower sample size or influence of human breeding activities (inbreeding, population bottlenecks). The high number of unique and singleton haplotypes detected in the studied breeds is in concordance with results of Kantanen et al. for the Serbian Podolian [19]. Moreover, modern cattle populations and also wild cattle have high haplotype diversity with predominance of haplogroups T1, T2 and T3 [8,69,70].

The mismatch analysis and the two statistically significant neutrality tests and non-significant raggedness and SDD indices applied to the mtDNA D-loop sequences support a demographic expansion of all studied breeds. However the absence of the star-like pattern with a highly dominant haplotypes among the detected haplotypes in the MJ network (Fig 5) does not support population expansion. These controversial results might be the result of short term of possible demographic expansion, or there are still not enough mutation accumulated in the gene.

### Management implications

The current study on molecular characterization of local breeds can contribute to the conservation of the Romanian Grey breed. The results obtained within the small population of endangered Romanian Grey cattle proves the availability of high genetic variability and highlights the relevance of the breed in forming of the overall genetic cattle biodiversity. The high genetic variability found might be the outcome of low selective pressure for production traits. Therefore, the genetic improvement of those productive performances could be a serious threat in the process of preservation the genetic diversity of Romanian Grey breed and must be avoided. In order to conserve the few remaining animals some major measures must be applied. We recommend that immediate actions to be taken and proper allocation of financial resources to be made available in order to conserve the valuable genetic variation of the Romanian Grey cattle breed, which still represents a reservoir of genetic diversity. Further, other priority measures like semen storage and artificial insemination could be applied in order to preserve the autochthonous Romanian Grey breed. Saving the Romanian Grey breed from extinction will be in benefits of both the Romanian agriculture and the international genetic heritage.

## Conclusions

A surprisingly high level of genetic diversity was found in the endangered Romanian Grey cattle population. All of our results confirmed that the breed’s genetic diversity is preserved correctly through the application of the current conservation program, however the number of the Romanian Grey individuals is extremely low and needs to be urgently increased. Results of the present study can doubtlessly help in improving the present conservation program in order to preserve the genetic diversity and protect the Romanian Grey breed from genetic loss. Furthermore, the results contribute to the general knowledge on genetic diversity found in Eastern European cattle breeds and could prove a valuable tool for the conservation efforts of animal genetic resources.

In order to design proper and effective conservation strategies, further attention and precise knowledge about all existing animals have to be taken into account. Therefore, urgent further studies, with other part of the mitochondrial genome (maternal side) and Y chromosome (paternal side), higher number of microsatellites, ancient DNA analysis or to involve all existent animals from the Romanian Grey breed, are recommended that ensure a better understanding of the actual genetic structure and past history of these studied breed.

## Supporting Information

S1 Table The collection sites and geographic coordinates of the Romanian breeds used in the study. (DOC)

S2 Table The haplotypes of studied breeds detected in this study and variable nucleotide positions in relation to a reference sequence from GenBank, accession no. V00654. (PDF)

S3 Table List of the cattle mtDNA sequences from Genbank used in this study. Sequences were downloaded from GenBank.
(XLSX)
