# Supplementary material for: Microsatellite and Mitochondrial DNA Study of Native Eastern European Cattle Populations: The Case of the Romanian Grey
Source: PLoS One. 2015 Sep 23;10(9):e0138736. doi: 10.1371/journal.pone.0138736 (PMC4580412; doi:10.1371/journal.pone.0138736)
Supplement: S1 Table — (DOC) [file pone.0138736.s001.doc]

**Table S1. The collection sites and geographic coordinates of the Romanian breeds used in the study.**

| **Breed** | **Number of samples** | **Collection sites** | **Geographic coordinates** |
| --- | --- | --- | --- |
| Romanian Grey | 6 | Bucuresti | 44°35'19.2"N 26°04'28.3"E |
|  | 16 | Dancu | 47°09'06.7"N 27°38'36.4"E |
|  | 5 | Piatra Neamt | 46°55'01.7"N 26°24'35.9"E |
|  | 5 | Pardina | 45°18'31.4"N 28°57'27.8"E |
| Romanian Brown | 19 | Arad | 46°10'15.8"N 21°14'08.6"E |
| Romanian Spotted | 5 | Targu Mures | 46°34'54.5"N 24°37'51.2"E |
|  | 2 | Ghilad | 45°27'56.2"N 21°08'38.9"E |
|  | 2 | Calacea | 46°41'01.6"N 21°56'32.5"E |
|  | 2 | Chisineu Cris | 46°30'49.8"N 21°30'32.1"E |
|  | 4 | Arad | 46°10'15.8"N 21°14'08.6"E |
| Romanian Black and White | 8 | Balotesti | 44°37'11.0"N 26°04'10.7"E |
|  | 4 | Nadlac | 46°29'22.4"N 21°30'14.9"E |
|  | 3 | Ghilad | 45°27'56.2"N 21°08'38.9"E |
