# Supplementary material for: Microsatellite and Mitochondrial DNA Study of Native Eastern European Cattle Populations: The Case of the Romanian Grey
Source: PLoS One. 2015 Sep 23;10(9):e0138736. doi: 10.1371/journal.pone.0138736 (PMC4580412; doi:10.1371/journal.pone.0138736)
Supplement: S2 Table — (PDF) [file pone.0138736.s002.pdf]

Table S2. The haplotypes of studied breeds detected in this study and variable nucleotide positions in relation to a reference sequence from GenBank, accession no. V00654.

Haplotype	No. of haplotype	Breeds	Haplogroup	Nucleotide positions and differences from ref. seq. V00645.1																																																															
				15921	15938	15939	15954	15972	15975	15988	15995	16004	16008	16016	16019	16022	16042	16045	16048	16049	16050	16054	16055	16057	16058	16062	16068	16074	16076	16084	16085	16092	16110	16113	16119	16121	16122	16126	16131	16133	16135	16139	16141	16154	16164	16185	16195	16200	16231	16232	16247	16248	16255	16260	16264	16294	16301	16302	16326	8	35	57	106	113	119	163	166	169	173	190
				**G**	**A**	**G**	**G**	**A**	**A**	**A**	**A**	**T**	**A**	**C**	**T**	**G**	**T**	**A**	**A**	**C**	**C**	**A**	**T**	**G**	**C**	**A**	**T**	**T**	**A**	**T**	**T**	**A**	**C**	**T**	**T**	**G**	**T**	**T**	**T**	**T**	**T**	**C**	**T**	**A**	**T**	**G**	**A**	**G**	**C**	**C**	**C**	**C**	**T**	**C**	**G**	**T**	**C**	**G**	**G**	**G**	**T**	**T**	**T**	**G**	**C**	**G**	**A**	**A**	**A**	**C**
Hap 1	1	RG	T3	.	.	.	C	.	.	.	.	.	.	T	.	.	.	.	.	.	.	.	.	.	.	.	.	.	.	C	.	G	.	.	.	.	.	.	.	.	.	.	.	.	.	A	.	.	.	.	.	.	.	.	.	.	.	.	.	.	.	.	C	.	.	A	.	G	.	.
Hap 2	6	RB	T3	.	.	.	.	.	.	.	.	.	.	T	.	.	.	.	.	.	.	.	.	.	.	.	.	.	.	C	.	.	.	.	.	.	.	C	.	.	.	.	.	.	.	.	.	.	.	.	.	.	C	.	.	.	.	.	.	.	.	.	.	.	.	.	.	.	.	.
Hap 3	1	RS	T3	.	.	.	.	.	.	.	.	.	.	T	.	.	.	.	.	.	.	.	.	.	.	.	.	.	.	C	.	.	.	.	.	.	.	.	.	.	.	.	C	.	.	.	.	.	.	.	.	.	.	.	.	.	.	.	.	.	.	.	.	.	.	A	.	.	.	.
Hap 4	1	RS	T3	.	.	.	.	C	T	.	.	.	.	T	.	.	.	.	.	.	.	.	.	.	.	.	.	.	.	C	.	.	.	.	.	.	.	.	.	.	.	.	.	.	.	.	.	.	.	.	.	.	.	.	.	.	.	.	.	.	.	.	.	.	.	A	.	G	.	.
Hap 5	1	RB	T3	.	.	.	.	.	.	.	.	.	.	T	.	.	.	.	.	.	.	.	.	.	.	.	.	.	.	C	.	G	.	.	.	.	.	.	.	.	.	.	.	.	.	.	.	.	.	.	.	.	.	.	.	.	.	.	.	.	.	.	.	.	.	A	.	G	.	.
Hap 6	4	RG, RB, RS	T3	.	.	.	.	.	.	.	.	.	.	T	.	.	.	.	.	.	.	.	.	.	.	.	.	.	.	C	.	.	.	.	.	.	.	.	.	.	.	.	.	.	.	.	.	.	.	.	.	.	.	.	.	.	.	.	.	.	.	.	.	.	.	A	.	G	.	.
Hap 7	1	RS	**T2**	.	.	.	.	.	.	.	.	.	.	T	.	.	.	.	.	.	.	.	.	C	.	.	.	.	.	C	.	.	.	.	.	.	.	.	.	.	.	.	.	.	.	A	.	.	.	.	.	.	C	.	.	.	.	.	.	.	.	.	.	.	.	A	.	.	.	.
Hap 8	2	RG	**T1**	.	.	.	.	.	.	.	.	.	.	T	.	.	.	.	.	.	.	.	.	.	.	.	.	.	.	C	C	.	.	.	.	.	.	.	C	.	.	.	.	.	.	.	.	.	.	.	.	.	.	.	.	.	.	.	.	.	.	.	.	.	.	A	G	.	.	.
Hap 9	1	RBW	T3	.	.	.	.	.	.	.	.	.	.	T	.	.	.	.	.	.	.	.	.	.	.	.	.	.	.	C	.	.	.	.	.	.	.	.	.	.	.	.	.	.	.	.	G	.	.	.	.	.	.	.	.	.	.	.	.	.	.	.	.	.	.	A	.	.	.	.
Hap 10	2	RS, RBW	T3	.	.	.	.	.	.	.	.	.	.	T	.	.	.	.	.	.	.	.	.	.	.	.	.	.	.	C	.	.	.	.	.	.	C	.	.	.	.	.	.	.	.	.	.	.	.	.	.	.	.	.	.	.	.	.	.	.	.	.	.	.	.	A	.	.	.	.
Hap 11	1	RBW	T3	.	.	.	.	.	.	.	.	.	.	T	.	.	.	.	.	.	.	.	.	.	.	.	.	.	.	C	.	.	.	.	.	.	.	.	.	.	.	T	.	.	.	.	.	.	.	.	T	.	.	.	.	.	.	.	.	.	.	.	.	.	.	A	.	G	G	.
Hap 12	1	RBW	T3	.	.	.	.	.	.	.	.	.	.	T	.	.	C	.	.	.	.	.	.	.	.	.	.	.	.	C	.	.	.	.	.	.	.	.	.	.	.	.	.	.	.	.	.	.	.	.	.	.	.	.	.	.	.	.	.	.	.	.	.	.	.	A	.	.	G	.
Hap 13	1	RBW	T3	.	.	.	.	.	.	.	.	.	.	T	.	.	.	.	.	.	.	.	.	.	.	.	.	.	G	C	.	.	.	.	.	A	.	.	.	.	.	.	.	.	.	.	.	.	.	.	.	.	.	.	.	.	.	.	.	.	.	.	.	.	.	A	.	.	.	.
Hap 14	1	RS	T3	.	.	.	.	.	.	.	.	.	.	T	.	.	.	.	.	.	.	.	.	.	.	.	.	C	.	C	.	.	.	.	.	.	.	.	.	.	.	.	.	.	.	.	.	.	.	.	.	.	.	.	.	.	.	.	.	.	.	C	.	.	.	A	.	.	.	.
Hap 15	1	RS	T3	.	.	.	.	.	.	.	.	.	.	T	.	A	.	.	.	.	.	.	.	.	.	.	.	C	.	C	.	.	.	.	.	.	.	.	.	.	.	.	.	.	.	.	.	.	.	.	.	.	.	.	.	.	.	.	.	.	.	.	.	.	.	A	G	G	.	T
Hap 16	2	RB	T3	.	.	.	.	.	.	.	.	.	.	T	.	.	.	.	G	.	.	.	.	.	.	.	.	.	.	C	.	.	.	.	.	.	.	.	.	.	.	.	.	.	.	.	.	.	.	.	.	.	.	.	.	.	.	.	.	.	.	.	C	.	.	A	.	.	.	.
Hap 17	1	RG	T3	.	.	.	.	.	.	.	.	.	.	T	.	.	.	G	.	.	.	.	C	.	.	.	.	.	.	C	.	.	.	.	.	.	.	.	.	.	.	.	.	.	.	.	.	.	.	.	.	.	.	.	.	.	.	.	.	.	.	.	.	.	.	A	.	.	.	.
Hap 18	1	RBW	T3	.	.	.	.	.	.	.	G	.	.	T	.	.	.	.	.	.	.	.	.	.	.	.	.	.	.	C	.	.	.	.	.	.	.	.	.	.	.	.	.	.	.	.	.	.	.	.	.	.	.	.	.	.	.	.	.	A	.	.	.	.	.	A	.	G	.	.
Hap 19	1	RBW	T3	.	.	.	.	.	.	.	.	.	.	T	.	.	.	.	.	.	.	.	.	.	.	.	.	.	.	C	.	.	.	.	.	.	.	.	.	.	.	.	.	.	.	.	.	.	.	T	.	.	.	.	.	.	.	.	.	.	.	.	.	.	.	A	.	.	.	.
Hap 20	1	RBW	T3	.	.	.	.	.	.	.	.	.	.	T	.	.	.	.	.	.	.	.	.	.	.	.	.	.	.	C	.	.	.	.	C	.	.	.	.	.	.	.	.	.	.	.	.	.	.	.	.	.	.	.	.	.	.	.	.	.	.	.	.	.	.	A	.	.	.	.
Hap 21	1	RBW	T3	.	.	.	.	.	.	.	.	.	.	T	.	.	.	.	.	.	.	.	.	.	.	.	.	.	.	C	.	.	.	.	.	.	.	.	.	.	.	.	.	.	.	.	.	A	.	.	.	.	.	.	.	.	.	.	.	.	.	.	.	.	.	A	.	.	.	.
Hap 22	1	RS	**T1**	.	.	.	.	.	.	G	.	.	.	T	.	.	.	.	.	.	T	.	.	.	.	.	.	.	.	C	.	.	.	.	.	.	.	.	.	.	.	.	.	.	.	.	.	.	.	.	.	.	.	.	.	.	.	.	.	.	.	.	.	.	.	A	.	G	.	.
Hap 23	1	RS	T3	.	.	.	.	.	.	.	.	.	.	T	C	.	.	.	.	.	.	.	.	.	.	.	.	.	.	C	.	.	.	.	.	.	C	.	.	.	.	.	.	.	.	.	.	.	.	.	.	.	.	.	.	.	.	.	.	.	.	.	.	.	.	A	.	G	.	.
Hap 24	1	RG	T3	.	.	.	.	.	.	.	.	.	.	T	.	A	.	.	.	.	.	.	.	.	.	G	.	.	.	C	.	.	.	.	.	.	.	.	.	.	C	.	.	.	.	.	.	.	.	.	.	.	.	.	.	.	.	.	.	.	.	.	.	.	.	A	.	G	.	.
Hap 25	1	RG	T3	.	.	.	.	.	.	.	.	.	.	T	.	.	.	.	.	.	.	.	.	.	.	.	.	.	.	C	.	.	.	.	.	.	.	.	.	.	.	.	.	.	.	.	.	.	.	.	.	.	.	.	.	.	.	.	.	A	.	.	C	.	.	A	.	G	.	.
Hap 26	2	RG	T3	.	.	.	.	.	.	.	.	.	.	T	.	A	.	.	.	.	.	.	.	.	.	.	.	.	.	C	.	.	.	.	.	.	.	.	.	.	.	.	.	.	.	.	.	.	.	.	.	.	.	.	.	.	T	.	.	.	.	.	.	.	.	.	.	G	.	.
Hap 27	2	RG	T3	.	.	.	.	.	.	.	.	.	.	T	.	A	.	.	.	.	.	.	.	.	.	.	.	.	.	C	.	.	.	.	.	.	.	.	.	.	.	.	.	.	.	.	.	.	.	.	.	.	.	.	.	.	.	.	.	.	.	.	.	.	.	.	.	G	.	.
Hap 28	1	RBW	T3	.	.	.	.	.	.	.	.	C	.	T	.	.	.	.	.	.	.	.	.	.	.	.	.	.	.	C	.	.	.	.	.	.	.	.	.	.	.	.	.	.	.	.	.	.	.	.	.	.	.	.	.	.	.	.	.	.	.	.	.	.	.	A	.	G	.	.
Hap 29	1	RS	T3	.	.	.	.	.	.	.	.	.	.	T	.	.	.	.	.	.	.	.	C	.	.	.	.	.	.	C	.	.	.	.	.	.	.	.	.	.	.	.	.	.	.	.	.	.	.	.	.	.	.	.	.	.	.	.	.	.	.	.	.	.	.	A	.	G	.	.
Hap 30	3	RB	T3	.	.	.	.	.	.	.	.	.	.	T	.	.	.	.	.	.	.	.	.	.	.	.	.	.	.	C	.	.	.	.	.	.	.	.	.	.	.	.	.	.	.	.	.	.	.	.	.	T	.	.	.	.	.	.	.	.	.	.	.	.	.	A	.	G	.	.
Hap 31	1	RG	T3	.	.	.	.	.	.	.	.	.	.	T	.	A	.	.	.	.	.	.	.	.	.	.	.	.	.	C	.	.	.	.	.	.	.	.	.	.	.	.	.	.	.	.	.	.	.	.	.	.	.	.	.	.	.	.	.	.	.	.	.	.	.	A	.	G	.	.
Hap 32	1	BR	T3	.	.	.	.	.	.	.	.	.	.	.	.	.	.	.	.	.	T	.	.	.	.	.	.	.	.	C	.	.	.	C	.	A	.	.	.	C	.	.	.	.	.	.	.	.	.	.	.	.	C	.	.	.	.	.	.	.	.	.	.	.	.	A	.	G	.	.
Hap 33	1	RG	T3	.	.	.	.	.	.	.	.	.	G	T	.	.	.	.	.	.	.	T	.	.	.	.	.	.	.	C	.	.	T	.	.	.	.	.	.	.	.	.	.	.	.	.	.	.	.	.	.	.	.	.	.	.	T	.	.	.	.	.	.	.	.	A	.	G	.	.
Hap 34	1	RG	T3	.	C	.	.	.	.	.	.	.	G	T	.	.	.	.	.	.	.	T	.	.	.	.	.	.	.	C	.	.	T	.	.	.	.	.	.	.	.	.	.	.	.	.	.	.	.	.	.	.	.	.	.	.	T	.	.	.	.	.	.	.	.	A	.	G	.	.
Hap 35	1	RBW	T3	.	.	.	.	.	.	.	.	.	.	T	.	.	.	.	.	.	.	.	.	.	.	.	.	.	.	C	.	.	.	.	.	.	.	.	.	.	.	.	.	.	.	.	.	.	.	.	.	.	.	.	.	.	.	.	A	.	G	.	.	A	.	A	.	G	.	.
Hap 36	2	RB	T3	.	.	.	.	.	.	.	.	.	.	T	.	.	.	.	.	.	.	.	.	.	.	.	C	.	.	C	.	.	.	.	.	.	.	.	.	.	.	.	.	.	C	.	.	.	.	.	.	.	.	.	.	.	.	A	.	.	.	.	.	.	.	A	.	G	G	.
Hap 37	8	RG	**T2**	.	.	.	.	.	.	.	.	.	.	T	.	.	.	.	.	.	.	.	.	C	.	.	.	.	.	C	.	.	.	.	.	.	.	.	.	.	.	.	.	.	.	A	.	.	.	.	.	.	C	.	.	.	.	.	.	.	.	.	C	.	.	A	.	G	.	.
Hap 38	1	RG	**T2**	.	.	A	.	.	.	.	.	.	.	T	.	.	.	.	.	.	.	.	.	C	.	.	.	.	.	C	.	.	.	.	.	.	.	.	.	.	.	.	.	.	.	A	.	.	.	.	.	.	C	.	.	.	.	.	.	.	.	.	C	.	.	A	.	G	.	.
Hap 39	1	RS	**T2**	.	.	.	.	.	.	.	.	.	.	T	.	.	.	.	.	T	.	.	.	C	.	.	.	.	.	C	.	.	.	.	.	.	.	.	.	.	.	.	.	.	.	A	.	.	.	.	.	.	C	.	.	.	.	.	.	.	.	.	.	.	.	A	.	G	.	.
Hap 40	1	RG	**T2**	.	.	.	.	.	.	.	.	.	.	T	.	.	.	.	.	.	.	.	.	C	T	.	.	.	.	C	.	.	.	.	.	.	.	.	.	.	.	.	.	.	.	A	.	.	.	.	.	.	C	.	.	.	.	.	.	.	.	.	.	.	.	A	.	G	.	.
Hap 41	1	RBW	**T2**	.	.	.	.	.	.	.	.	.	.	T	.	.	.	.	.	.	.	.	.	C	.	.	.	.	.	C	.	.	.	.	.	.	.	.	.	.	.	.	.	.	.	A	.	.	.	.	.	.	C	.	.	.	.	.	.	.	.	.	.	.	.	A	.	G	.	.
Hap 42	2	RS	T3	.	.	.	.	.	.	.	.	.	.	T	.	.	.	.	.	.	.	.	.	.	.	.	.	.	.	C	.	.	.	.	.	.	.	.	.	.	.	.	.	.	.	.	.	.	.	T	.	.	.	.	A	.	.	.	.	.	.	.	.	.	.	A	.	G	.	.
Hap 43	1	RBW	T3	.	.	.	.	.	.	.	.	.	.	T	.	.	.	.	.	.	.	.	.	.	.	.	.	.	.	C	.	.	.	.	.	.	.	.	.	.	.	.	.	.	.	.	.	.	.	T	.	.	.	.	.	.	.	.	.	A	.	.	.	.	.	A	.	G	.	.
Hap 44	3	RG	T3	.	.	.	.	.	.	.	.	.	.	T	.	.	.	.	.	.	.	.	.	.	T	.	.	.	.	C	.	.	.	.	.	.	.	.	.	.	.	.	.	G	.	.	.	.	.	.	.	.	.	.	.	C	.	.	.	.	.	.	.	.	.	A	.	G	.	.
Hap 45	1	RG	T3	.	.	.	.	.	.	.	.	.	.	T	.	.	.	.	.	.	.	.	.	.	T	.	.	.	.	C	.	.	.	.	.	.	.	.	.	.	.	.	.	G	.	.	.	.	.	.	.	.	.	.	.	C	.	.	.	.	.	.	.	.	T	A	.	G	.	.
Hap 46	2	RBW	T3	.	.	A	.	.	.	.	.	.	.	T	.	.	.	.	.	.	.	.	.	.	.	.	.	.	.	C	.	.	.	.	.	.	.	.	.	.	.	.	.	.	.	.	.	.	.	.	.	.	.	A	.	.	T	.	.	.	.	.	.	.	.	A	.	.	.	.
Hap 47	1	RS	T3	.	.	.	.	.	.	.	.	.	.	T	.	.	.	.	.	.	.	.	.	.	.	.	.	.	.	C	.	.	.	.	C	.	.	.	.	.	.	.	.	.	.	.	.	.	.	.	.	.	.	.	.	.	.	.	.	.	.	.	.	.	.	A	.	G	.	.
Hap 48	1	RB	T3	A	.	.	.	.	.	.	.	.	.	T	.	.	.	.	.	.	.	.	.	.	.	.	.	.	.	C	.	.	.	.	.	.	.	.	.	.	.	.	.	.	.	.	.	.	.	.	.	.	.	.	.	.	.	.	.	.	.	.	.	.	.	A	.	G	.	.
Hap 49	2	RG	T3	.	.	.	.	.	.	.	.	.	.	T	.	.	.	.	.	.	.	.	.	.	.	.	.	.	.	C	.	.	.	.	.	.	.	.	.	.	.	.	.	.	.	.	.	.	.	.	.	.	.	.	.	.	.	.	.	.	.	.	.	.	T	A	.	G	.	.
Hap 50	1	RS	T3	.	.	.	.	.	.	.	.	.	.	T	.	.	.	.	.	.	.	.	.	.	.	.	.	.	.	C	.	.	.	.	.	.	.	.	.	.	.	.	.	.	.	.	.	.	T	.	.	.	.	.	.	.	.	.	.	.	.	.	.	.	.	A	.	G	.	.
Hap 51	2	RG, RB	T3	.	.	.	.	.	.	.	.	.	.	T	.	.	.	.	.	.	.	.	.	.	.	.	.	.	.	C	.	.	.	.	.	.	.	.	.	.	.	.	.	.	.	.	.	.	.	.	.	.	.	.	.	.	.	.	.	.	.	.	.	.	.	A	.	.	.	.
Hap 52	1	RB	T3	.	.	.	.	.	.	.	.	.	.	T	.	.	.	.	.	.	.	.	.	.	.	.	.	.	.	C	.	.	.	.	.	.	.	.	.	.	.	.	.	.	.	.	.	.	T	.	.	.	.	.	.	.	.	.	.	.	.	.	.	.	.	A	.	.	.	.

RG=Romanian Grey, RB=Romanian Brown, BS=Romanian Spotted, BRW=Romanian Black and White
